# Sex differences in inflammatory markers in patients hospitalized with COVID-19 infection: Insights from the MGH COVID-19 patient registry

**DOI:** 10.1371/journal.pone.0250774

**Published:** 2021-04-28

**Authors:** Emily S. Lau, Jenna N. McNeill, Samantha M. Paniagua, Elizabeth E. Liu, Jessica K. Wang, Ingrid V. Bassett, Caitlin A. Selvaggi, Steven A. Lubitz, Andrea S. Foulkes, Jennifer E. Ho

**Affiliations:** 1 From the Cardiovascular Research Center, Massachusetts General Hospital, Boston, MA, United States of America; 2 Division of Cardiology, Massachusetts General Hospital, Boston, MA, United States of America; 3 Division of Pulmonary and Critical Care, Massachusetts General Hospital, Boston, MA, United States of America; 4 Division Infectious Disease, Massachusetts General Hospital, Boston, MA, United States of America; 5 Mongan Institute, Massachusetts General Hospital, Boston, MA, United States of America; 6 Biostatistics Center of Massachusetts General Hospital, Boston, MA, United States of America; Heidelberg University Hospital, GERMANY

## Abstract

**Background:**

Men are at higher risk for serious complications related to COVID-19 infection than women. More robust immune activation in women has been proposed to contribute to decreased disease severity, although systemic inflammation has been associated with worse outcomes in COVID-19 infection. Whether systemic inflammation contributes to sex differences in COVID-19 infection is not known.

**Study design and methods:**

We examined sex differences in inflammatory markers among 453 men (mean age 61) and 328 women (mean age 62) hospitalized with COVID-19 infection at the Massachusetts General Hospital from March 8 to April 27, 2020. Multivariable linear regression models were used to examine the association of sex with initial and peak inflammatory markers. Exploratory analyses examined the association of sex and inflammatory markers with 28-day clinical outcomes using multivariable logistic regression.

**Results:**

Initial and peak CRP were higher in men compared with women after adjustment for baseline differences (initial CRP: ß 0.29, SE 0.07, p = 0.0001; peak CRP: ß 0.31, SE 0.07, p<0.0001) with similar findings for IL-6, PCT, and ferritin (p<0.05 for all). Men had greater than 1.5-greater odds of dying compared with women (OR 1.71, 95% CI 1.04–2.80, p = 0.03). Sex modified the association of peak CRP with both death and ICU admission, with stronger associations observed in men compared with women (death: OR 9.19, 95% CI 4.29–19.7, p <0.0001 in men vs OR 2.81, 95% CI 1.52–5.18, p = 0.009 in women, P_interaction_ = 0.02).

**Conclusions:**

In a sample of 781 men and women hospitalized with COVID-19 infection, men exhibited more robust inflammatory activation as evidenced by higher initial and peak inflammatory markers, as well as worse clinical outcomes. Better understanding of sex differences in immune responses to COVID-19 infection may shed light on the pathophysiology of COVID-19 infection.

## Introduction

Emerging data suggest that men are at higher risk for severe coronavirus disease 2019 (COVID-19) infection compared with women despite similar rates of infection [1]. Worldwide, men accounted for approximately 60% of deaths attributed to COVID-19 [2]. Many theories have been proposed to explain these observations including the protective role of estrogen, ACE2 gene expression on the X chromosome, and more robust immune activation in women [3, 4]. Epidemiologic studies have consistently demonstrated more exuberant immune responses in women compared with men. For example, women more frequently report severe local and systemic reactions and have been shown to generate more robust antibody responses in response to the influenza vaccine compared with men [5]. Moreover, the prevalence of autoimmune disease is far higher in women than men in the general population [6]. Finally, sex differences in inflammatory markers including hsCRP and IL-6 and have been described [7–9]. Despite a growing body of evidence supporting sex differences in immune response, how inflammation contributes to COVID-19 disease severity in men vs women is not known. In light of reports implicating systemic inflammation as a potential driver of severity of COVID-19 disease, it is notable that stronger immune responses in women have been postulated to contribute to decreased mortality in women [10].

In this context, we sought to investigate the role of systemic inflammation in contributing to biologic sex differences among patients hospitalized with COVID-19 infection in the Massachusetts General Hospital COVID-19 Registry, a comprehensive repository of observational data from COVID-19 PCR positive patients. Our study aims are twofold. First, we sought to investigate the association of biologic sex with pro-inflammatory markers among 781 patients hospitalized with confirmed COVID-19 at Massachusetts General Hospital. Second, we examined whether sex modifies the association of inflammatory biomarkers with clinical outcomes in hospitalized patients with COVID-19 infection. We define biologic sex as the sex that an individual was assigned at birth and will refer to sex as a binary variable (male/men vs female/women) for ease of description, acknowledging that this terminology does not capture the complexity of sex- and gender-based biology [11].

## Materials & methods

### Study population

This retrospective observational study included adults with confirmed COVID-19 infection who met criteria for inpatient admission at the Massachusetts General Hospital between March 8 to April 27, 2020 [12]. We excluded patients with active cancer except non-melanoma skin cancers (n = 35), current pregnancy (n = 19), age < 18 years (n = 7), and those with missing lab values or covariates (n = 22), yielding a final sample of 781 patients. Patient data were obtained via manual chart review of electronic health records and data extractions via the Partners Enterprise Data Warehouse. Approval for this study as a minimum personal health information (PHI) study was obtained by the Partners Institutional Review Board. Written informed consent was obtained for patients who provided samples for the Partners Biobank only (protocol 2009P002312). The remaining participants did not require informed consent as no samples were stored and no additional data were ascertained.

### Statistical analysis

Baseline demographics and clinical characteristics were summarized for men and women separately. Results are reported as means ± standard deviation (SD) or medians and inter-quartile ranges (IQR) for continuous variables and as percentages for dichotomous variables. Differences between men and women were tested using Chi square, Student’s t-test, or Wilcoxon rank sum test as appropriate. Inflammatory markers of interest including C-reactive protein (CRP), erythrocyte sedimentation rate (ESR), interleukin-6 (IL-6), procalcitonin (PCT), D-dimer, and ferritin, were obtained for clinical indications. Initial inflammatory markers refer to the first inflammatory markers obtained upon hospital admission, and peak inflammatory markers refer to the highest value obtained during hospital admission. Inflammatory markers were natural log-transformed due to right-skewed distributions. We examined the association of sex with natural log-transformed initial and peak inflammatory markers using multivariable linear regression models, adjusted for age, body mass index (BMI), hypertension (HTN), diabetes mellitus (DM), cardiovascular, pulmonary, liver, and kidney disease, smoking status, non-steroidal anti-inflammatory drug (NSAID) use, statin use, and immunosuppressant use.

In exploratory analyses, we examined the association of inflammatory markers with clinical outcomes in both sex-pooled and sex-stratified models using multivariable logistic regression models. Clinical outcomes of interest included ICU admission, death during the index admission, and the composite of ICU admission or death. Outcomes were ascertained up to 28 days after presentation to care (defined as the first encounter with the health system) until discharge or death. Specifically, we examined sex*inflammatory marker interaction terms to test whether sex modified the association of inflammatory markers and outcomes in sex-pooled models, using multivariable models adjusted for age, BMI, HTN, DM, cardiovascular, pulmonary, liver, and kidney disease, smoking status, and performed sex-stratified analyses to evaluate the association in men and women, separately.

Analyses were conducted using SAS version 9.4 (SAS Institute, Cary, NC) and STATA version 15.1 (College Station, TX). All tests were two-sided and a p-value of <0.05 was considered significant.

## Results

Of 781 individuals, 453 (58%) were men and 328 (42%) were women (**Table 1**). Men and women were of similar age and had similar BMI. Men were more likely to be current or former smokers and to have kidney disease, while women were more likely to have a history of autoimmune disease and asthma. Men were more frequently taking statins at baseline and more likely to receive empiric treatment with hydroxychloroquine during their hospitalization.

**Table 1:** Baseline characteristics, inflammatory markers, and outcomes in men and women with COVID-19 infection.

	Men (N = 453)		Women (N = 328)	
**Demographics**				
**Age, years**	61 (17)		62 (18)	
**White race, n (%)**	179 (41%)		136 (42%)	
**Hispanic ethnicity, n (%)**	159 (36%)		120 (37%)	
**Body mass index, kg/m^2^**	30.5 (11.2)		30.8 (7.3)	
**Comorbidities**				
**Cardiovascular disease, n (%)**	118 (26%)		67 (20%)	
**Type 2 diabetes, n (%)**	168 (37%)		115 (35%)	
**Hypertension, n (%)**	238 (53%)		178 (54%)	
**Pulmonary disease, n (%)**	137 (30%)		108 (33%)	
**COPD, n (%)**	53 (12%)		37 (11%)	
**Asthma, n (%)**	51 (11%)		55 (17%)*	
**Obstructive sleep apnea, n (%)**	29 (6%)		22 (7%)	
**Autoimmune disease, n (%)**	32 (7%)		44 (14%)*	
**Kidney disease, n (%)**	95 (21%)		42 (13%)*	
**Liver disease, n (%)**	39 (9%)		33 (10%)	
**Current cigarette smoker, n (%)**	47 (10%)		13 (4%)	
**Former cigarette smoker, n (%)**	178 (39%)		79 (24%)^†^	
**Baseline medications**				
**Immunosuppressants, n (%)**	29 (6%)		25 (8%)	
**NSAIDs, n (%)**	96 (21%)		77 (24%)	
**Statins, n (%)**	214 (47%)		130 (40%)*	
**ACE inhibitors, n (%)**	71 (16%)		53 (16%)	
**Empiric therapy**				
**Remdesevir, n (%)**	4 (1%)		2 (1%)	
**Tocilizumab, n (%)**	5 (1%)		4 (1%)	
**Hydroxychloroquine, n (%)**	231 (51%)		127 (39%)^†^	
**Azithromycin, n (%)**	207 (46%)		136 (42%)	
**Inflammatory Markers**	**Initial**	**Peak**	**Initial**	**Peak**
**CRP, mg/L**	76 (38–145)	145 (74–263)	59 (27–136)*	125 (54–211)^†^
**ESR, mm/h**	35 (21–57)	59 (32–110)	40 (27–59)*	54 (35–91)
**Procalcitonin, ng/mL**	0.16 (0.10–0.31)	0.18 (0.10–0.53)	0.12 (0.08–0.22)^†^	0.13 (0.08–0.28)^†^
**Ferritin, ug/L**	631 (360–1230)	1027 (546–2142)	413 (224–675)^†^	562 (306–1049)^†^
**D-dimer, ng/mL**	921 (608–1683)	1749 (897–3944)	1063 (676–1760)	1587 (848–3174)
**IL-6, pg/mL**	37 (16–88)	40 (17–95)	24 (10–56)*	25 (11–58)*
**Symptoms**				
**Symptomatic at presentation**	435 (97%)		319 (98%)	
**Time from symptom onset to presentation, days**	5.4 (6.1)		5.5 (6.8)	
**Outcomes**				
**Death, n (%)**	73 (16%)		43 (13%)	
**ICU admission, n (%)**	152 (34%)		90 (28%)	
**ICU admission or death, n (%)**	179 (40%)		115 (35%)	

Values are means (standard deviations) or medians (inter-quartile ranges) unless otherwise noted.

* for p<0.05 and

^†^ for p<0.001. Abbreviations: ACE inhibitors = angiotensin converting enzyme inhibitors, BMI = body mass index, IVIg = intravenous immunoglobulin, NSAIDs = non-steroid anti-inflammatory drugs. Biomarker data were available for 665 participants.

Initial and peak inflammatory markers in men and women are displayed (**Table 1**). Initial CRP, IL-6, PCT and ferritin were higher in men, while initial ESR was higher in women. Peak inflammatory markers were higher in men excluding ESR and D-dimer. These observations persisted after multivariable adjustment, with significant associations between male sex and initial CRP, ferritin, and IL-6, as well as peak CRP, PCT, ferritin, and IL-6 (p<0.05 for all, **Table 2**). Initial ESR levels were significantly lower in men than women (ß -0.20, SE 0.08, p = 0.01), but there was no difference in peak ESR levels between men and women (p = 0.54).

**Table 2:** Sex as a predictor of initial and peak inflammatory markers.

Inflammatory Marker	Initial			Peak		
	ß^†^	SE	p-value	ß^†^	SE	p-value
CRP, mg/L	0.29	0.07	0.0001	0.31	0.07	<0.0001
ESR, mm/h	-0.20	0.08	0.01	0.05	0.08	0.54
Procalcitonin, ng/mL	0.12	0.08	0.11	0.19	0.08	0.01
D-dimer, ng/mL	-0.04	0.07	0.58	0.13	0.07	0.07
IL-6, pg/mL	0.36	0.13	0.005	0.35	0.13	0.007
Ferritin, ug/L	0.43	0.07	<0.0001	0.55	0.07	<0.0001

^†^ß-coefficient: regression coefficients represent difference between men and women (referent) for continuous log-transformed variables. Multivariable model for adjusts for age, body mass index, cardiovascular disease, hypertension, type 2 diabetes, liver disease, kidney disease, pulmonary disease, smoking status, non-steroidal anti-inflammatory drug use, statins, and immunosuppressants. Abbreviations: ESR = erythrocyte sedimentation rate, CRP = C-reactive protein, IL-6 = interleukin-6, SE = standard error.

**Fig 1:**
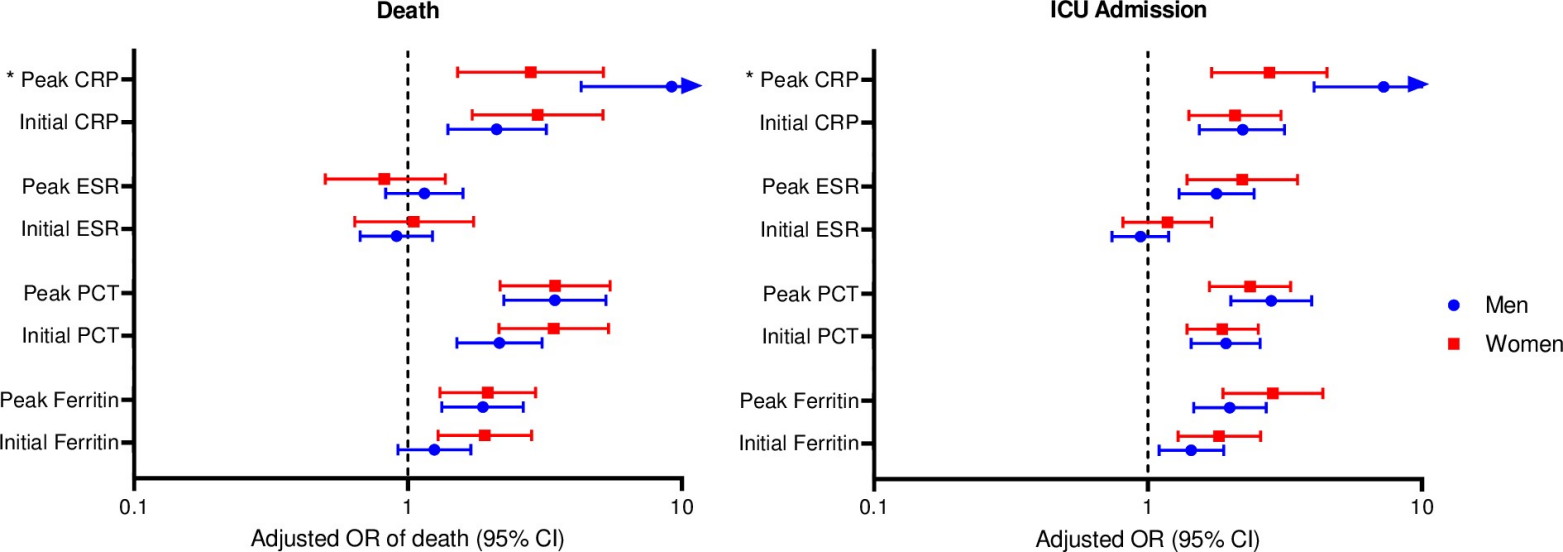
The association of inflammatory markers with death and ICU admission in men and women with COVID-19 infection. * = sex*inflammatory marker p_interaction_ <0.05.

Finally, men had more than 1.5-fold increased odds of dying compared with women even after multivariable adjustment (OR 1.71, 95% CI 1.04–2.80, p = 0.03). There was no difference in ICU admission or the composite of death and ICU admission between men and women (p>0.05). In inflammatory marker analyses, we found that sex modified the association of peak CRP with both death and ICU admission. A 1-SD higher peak CRP was associated with an over 9-fold increased odds of death in men compared with 2.8-fold increased odds among women (OR 9.19, 95% CI 4.29–19.7, p <0.0001 in men vs OR 2.81, 95% CI 1.52–5.18, p = 0.009 in women, P_interaction_ = 0.02, **Fig 1**). This interaction was also observed for ICU admission (OR 7.26, 95% CI 4.04–13.04 in men vs OR 2.78, 95% CI 1.71–4.51 in women, P_interaction_ = 0.009).

## Discussion

In a sample of 781 COVID-19 positive hospitalized patients, we show significant sex differences in inflammatory markers and outcomes including (1) higher levels of both initial and peak inflammatory markers in men compared with women, (2) men with COVID-19 infection had nearly 2-fold increased odds of dying compared with women, and (3) the association of peak CRP with death and ICU admission is more pronounced in men compared with women. These findings suggest that more robust inflammatory activation may contribute to greater COVID-19 disease severity in men.

The male bias in COVID-19 severity and mortality has been observed in nearly all 38 countries with publicly available sex-disaggregated data, with an associated risk of death 1.7 times higher in men compared with women [4, 13]. These data have been corroborated by numerous studies conducted worldwide [14–16], and are consistent with our findings that demonstrate significantly increased odds of death in men hospitalized with COVID-19 infection compared with women. While sociological factors likely contribute in part to the disproportionate disease severity observed in men with COVID-19 infection, the consistent sex differences observed worldwide highlight the importance of biologic risk determinants in mediating COVID-19 disease severity.

Biologic sex differences in innate and adaptive immune responses have been proposed to explain the male bias observed in COVID-19 infections. Gene expression of immune cell subsets demonstrate sex-specific patterns and regulation [17]. Moreover, sex chromosomes are also implicated in immune regulation, whereby incomplete X chromosome inactivation has been associated with female-biased autoimmune diseases and vaccine efficacy [18–20]. Finally, sex hormones including estrogen and testosterone also have direct effects on immune cell function [21, 22]. Collectively, these biologic sex differences in immunity suggest that men may be more vulnerable to COVID-19 infection due to lower immune responses, while women are potentially “protected” by a strong immune response.

Interestingly, emerging evidence do not offer support for the hypothesis that decreased immune responses contribute to severe COVID-19 infection in men. Instead, COVID-19 related morbidity and mortality appears to be mediated by exuberant viral stimulated inflammation, characterized by increased levels of inflammatory biomarkers and cytokines. In an early study of 548 COVID-19 inpatients in China, men had higher levels of hsCRP, ferritin, and IL-10, but lower lymphocyte count compared with women even after adjustment for age and comorbidities [23]. Other studies have found greater upregulation of pro-inflammatory cytokines including IL-7, IL-16, and IL-18 in men with COVID-19 infection compared with women [24]. Our findings are in line with prior studies: we show higher levels of initial CRP, ferritin, and IL-6, and peak CRP, PCT, ferritin, and IL-6 in men compared with women. Moreover, peak CRP levels were more strongly associated with death and ICU admission in men compared with women. Of note, baseline CRP and ESR concentrations are higher in women than in men in the general population, while plasma ferritin and IL-6 levels are higher in men. Taken together, our findings highlight the potential role of systemic inflammation in mediating COVID-19 disease severity and mortality, particularly in men.

This study has several limitations. First, ascertainment of inflammatory biomarkers was not standardized and may have been associated with the patients’ clinical condition. Specifically, peak biomarkers were drawn at different time points across the hospitalization and the frequency of blood draws was higher in men vs women for all biomarkers except PCT and IL-6, introducing potential ascertainment bias. Second, the study time period preceded routine use of dexamethasone, now considered standard of care for the treatment of patients with severe COVID-19 infection; routine dexamethasone use may have influenced our study results. Third, while inflammatory biomarker levels especially CRP are influenced by sex hormone levels, detailed reproductive history including menopausal and hormone status was not routinely collected on participants in the registry. Fourth, the study was conducted at a single academic medical center with a modest number of patients and may not be generalizable to other settings. Finally, this was an observational study, limiting causal inferences. Despite these limitations, our study is strengthened by a rigorously curated registry including extensive clinical and demographic histories, hospital admission labs, drugs administered during hospitalization, 28-day complications, and outcomes. Moreover, inclusion of consecutive patients admitted during the study period reduces sample selection bias.

## Conclusions

In sum, our findings show that men hospitalized with COVID-19 infection display higher levels of both initial and peak inflammatory markers compared with women. Compared with women, men had higher odds of death, and peak CRP levels were more strongly associated with both death and ICU admission in men vs women. Further understanding of inflammatory and immune responses to COVID-19 in men and women is vital for the development of targeted therapies.

## Abbreviations

ARDS: acute respiratory distress syndrome
BMI: body mass index
COVID-19: coronavirus disease 2019
DM: diabetes mellitus
ESR: erythrocyte sedimentation rate
CRP: C-reactive protein
HTN: hypertension
IL-6: interleukin-6
PCT: procalcitonin
SARS-CoV-2: severe acute respiratory syndrome coronavirus 2
